# Somatic Embryogenesis in Coffee: The Evolution of Biotechnology and the Integration of Omics Technologies Offer Great Opportunities

**DOI:** 10.3389/fpls.2017.01460

**Published:** 2017-08-21

**Authors:** Nádia A. Campos, Bart Panis, Sebastien C. Carpentier

**Affiliations:** ^1^Department of Biosystems, KU Leuven Leuven, Belgium; ^2^Bioversity International Leuven, Belgium; ^3^Facility for Systems Biology Based Mass Spectrometry, KU Leuven Leuven, Belgium

**Keywords:** totipotency, somatic embryogenesis, *Coffea arabica*, tissue culture, molecular biology, coffee

## Abstract

One of the most important crops cultivated around the world is coffee. There are two main cultivated species, *Coffea arabica* and *C. canephora.* Both species are difficult to improve through conventional breeding, taking at least 20 years to produce a new cultivar. Biotechnological tools such as genetic transformation, micropropagation and somatic embryogenesis (SE) have been extensively studied in order to provide practical results for coffee improvement. While genetic transformation got many attention in the past and is booming with the CRISPR technology, micropropagation and SE are still the major bottle neck and urgently need more attention. The methodologies to induce SE and the further development of the embryos are genotype-dependent, what leads to an almost empirical development of specific protocols for each cultivar or clone. This is a serious limitation and excludes a general comprehensive understanding of the process as a whole. The aim of this review is to provide an overview of which achievements and molecular insights have been gained in (coffee) somatic embryogenesis and encourage researchers to invest further in the *in vitro* technology and combine it with the latest omics techniques (genomics, transcriptomics, proteomics, metabolomics, and phenomics). We conclude that the evolution of biotechnology and the integration of omics technologies offer great opportunities to (i) optimize the production process of SE and the subsequent conversion into rooted plantlets and (ii) to screen for possible somaclonal variation. However, currently the usage of the latest biotechnology did not pass the stage beyond proof of potential and needs to further improve.

## Introduction

### Rationale

Coffee is one of the most important commodities cultivated worldwide and has a great economic impact in many countries, especially in South America (FAOSTAT, 2014). Although more than 130 different species belonging to the *Coffea* genus have been described, only two of them are mainly commercially exploited: *Coffea arabica* and *C. canephora*. There exist other species such as *C. iberica, C. dewevrei*, and *C. racemosa* that are thus far only cultivated to satisfy local markets (Kumar et al., 2006). The most cultivated variety for *C. canephora* is robusta. *C. arabica* has many different important cultivars and is responsible for 60% of the world production. It is considered to have superior beverage qualities compared to robusta. However, the production costs for *C. arabica* are much higher due to crop management practices, higher susceptibility toward diseases and the need for more stringent environmental conditions (less adapted to temperature changes, requirement of more rain or irrigation and prefers higher altitudes due to more mild temperatures) (van der Vossen et al., 2015). While *C. canephora* is a diploid species (2*n* = 2*x* = 22) and presents a higher diversity, *C. arabica* is tetraploid (2*n* = 4*x* = 44) and shows a very narrow genetic diversity attributed to its evolution and reproductive biology (self-pollination) (Lashermes et al., 1999, 2000). Moreover, coffee plants take 2 years to complete their life cycle. All these characteristics make classic genetic improvement a big challenge, taking at least 20 years to have a new genotype in the market (Etienne, 2005; Santana-Buzzy et al., 2007; Tonietto et al., 2012).

One powerful biotechnological tool used in crop improvement is somatic embryogenesis (SE). By SE we understand the production of an embryo from somatic tissues without fecundation (de Feria et al., 2003). Embryogenic cells show two important characteristics; they are able to multiply or to proliferate, which makes SE suitable for mass production of elite cultivars (de Feria et al., 2003), and the fact that plants can be regenerated from one single cell. The latter characteristic is essential for genetic engineering and somatic hybridization. Moreover, SE can also be used to conserve interesting genotypes and/or the ones that are threatened with extinction (Yang and Zhang, 2010). Because SE formation is based on cellular totipotency it has also been used as a model to investigate morphological, physiological, molecular and biochemical events that occur during the onset and development of embryogenesis in higher plants (Quiroz-Figueroa et al., 2006). In coffee, the most direct application of SE is the rapid multiplication of elite genotypes, specially hybrid heterozygous ones (Etienne et al., 2016).

The SE technology has been studied in coffee since 1970 (Staritsky, 1970). The methodologies to induce SE and the further development of embryos are genotype-dependent, which leads to an almost empirical development of specific protocols for each species (Santana-Buzzy et al., 2007). In the recent years, information about the molecular mechanisms of SE induction has been gained. In this review, we provide an overview about the general concepts of somatic embryogenesis and important molecular markers found in model plants, how this knowledge has been applied on coffee SE and how future knowledge could be applied to improve protocols. Additionally we introduce a new hypothesis about the acquisition of embryogenic capacity. We want to instigate researchers to see the classical concept from a different point of view and reflect about the type of explant that is used for SE induction.

### SE General Concepts

In general, somatic embryogenesis for all species is initiated by exposing plant tissues to the right stimulus, most often to plant hormones (Yang and Zhang, 2010). A right balance between the applied hormones and internal factors can induce the reprogramming of a differentiated somatic cell, but it could also promote the proliferation of totipotent undifferentiated cells that are dormant present in some tissues, being the plant stem cells. The “classical” theory about the formation of SE is that differentiated somatic cells can regain their embryogenesis capacity and be reprogrammed to differentiate into new viable embryos (Marsoni et al., 2008; Yang and Zhang, 2010). Irrespective of the initiation of the totipotency theory, somatic embryos can be obtained in two different ways, directly or indirectly. Following the direct way, the embryos are formed without intermediate callus formation (proliferation of cells) directly on the explant (leaves, roots or other part of plant). In the indirect way, first an embryogenic callus is formed and then embryos arise from this callus (Yang and Zhang, 2010). In the indirect way, two distinct phases are involved, called induction and expression. The induction stage is marked by changes in the metabolism and gene expression, leading to the differentiation into embryos in the expression phase (Fehér et al., 2003; Jiménez, 2005).

The use of growth regulators is practically essential to obtain somatic embryos in both processes, direct and indirect way. The optimal concentration, time of treatment and type of growth regulator varies according the species or even according the cultivars within the same species. Although essential to induction of SE process, auxins are negatively effecting embryo development. To solve this problem, most of protocols suggest dropping the concentration or even omitting auxins after the multiplication of totipotent cells to allow differentiation and consequent protoderm development. Associated periclinal cell divisions result in tissue invagination and establishment of the embryonic axis, being the beginning of embryo development (Toonen et al., 1994; van Boxtel and Berthouly, 1996; Simões-costa et al., 2009; Silva et al., 2015).

### Characteristics of General Embryogenic Cells

**FIGURE 1:**
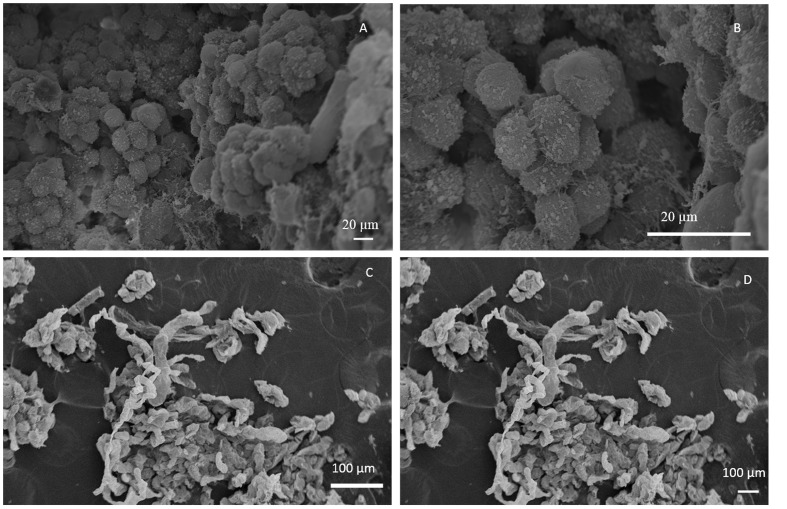
Scanning Electron Microscopy of coffee callus cells. **(A)** cells in the yellow friable callus arranged in clusters; **(B)** round cells in the yellow friable callus; **(C,D)** transparent watery callus with elongated cells. Adapted from ©Padua et al., 2014. This picture is being reproduced with permission from the copyright holders.

Embryogenic calli can be distinguished from non-embryogenic calli based on their morphological characteristics (Yang and Zhang, 2010; Padua et al., 2014; Silva et al., 2014). In general, embryogenic calli are yellow and friable and their cells are small, isodiametric, arranged in clusters, with a dense cytoplasm, a nucleus with salient nucleoli and rich in small amyloplasts. Non-embryogenic calli are spongy and translucent showing cells that are more elongated, with the vacuole occupying a big volume of the cytoplasm, higher number of vesicles and absence of cytoplasmic organelles (**Figure 1**) (Gatica-Arias et al., 2008; Padua et al., 2014; Silva et al., 2014). Zygotic and somatic embryo development processes for all plant species are quite similar. That is the reason why somatic embryogenesis is often used to study zygotic embryogenesis. Generally, morphogenetic and metabolic phases are distinguished. In the morphogenetic stage the structure of embryos are established and the second stage is marked by a pronounced biochemical activity. In dicots, the morphogenetic stage is divided in four phases; globular, heart, torpedo and cotyledonary stage (Etienne, 2005; Yang and Zhang, 2010; Etienne et al., 2013; Padua et al., 2014). Despite that both zygotic and somatic embryogenesis are similar, there are some differences in morphological and histochemical aspects during embryo development. The shape of somatic embryos are normally more irregular than zygotic embryos due to a different cell elongation and different storage components between both process (Bertrand et al., 2012; Etienne et al., 2013).

### Can Totipotency Be Induced from Differentiated Somatic Cells?

The “classical” concept for initiation of somatic embryogenesis affirm that molecular reprograming of somatic cells is essential. In this process, already differentiated cells regain the totipotency capacity (Fehér et al., 2003; Yang and Zhang, 2010) and under the right stimulus they can differentiate into new embryos. Although this is the most accepted theory about totipotency in SE, we would like to discuss an alternative hypothesis. We hypothesize that the cells that are able to differentiate into embryos don’t pass through the process of dedifferentiation. They are pools of meristematic cells that keep the totipotency throughout plant development and under the right stimulus they multiply and differentiate to form a new viable embryo. These cells would act then, as a plant stem cell. The fact that only a limited amount of cells are responsive to SE induction through the right stimulation is a point to strengthen our hypothesis (Quiroz-Figueroa et al., 2002; Yang and Zhang, 2010). Not matter the stimulation provided, some cells will never become an embryo. The responsive cells in our point of view are the ones that keep the embryogenic capacity and could be considered as plant stem cells. Explants currently used for direct SE induction are microspores, ovules, immature zygotic embryos, seedlings and young leaves (Yang and Zhang, 2010), all young tissues. Such young tissues relatively contain more stem cells. With aging, the ratio totipotent stem cells/differentiated cell decreases. Auxins are considered essential to induce the process of SE in the view of the general theory about totipotency capacity and it would be the induction of the redifferentiation and proliferation of the embryogenic cells. In our theory it is the breakage factor of ‘dormancy’ and the proliferation, being also essential to complete the SE process. This theory arose from our personal discussion during years of embryogenesis study. It is an invitation to see the classical concept of embryogenic acquisition from a different point of view and stimulate new researches in this topic.

## Coffee Somatic Embryogenesis

### General Overview

**FIGURE 2:**
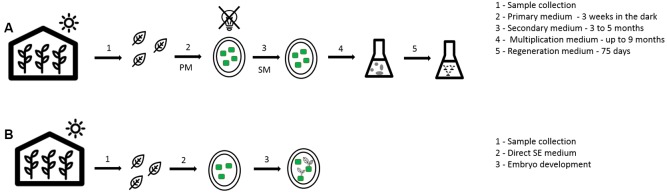
General overview of somatic embryogenesis in coffee. **(A)** High frequency method (indirect way); **(B)** low frequency method (direct way).

The first trails on coffee SE were executed using orthotropic shoots in different coffee species, with obtaining of embryos only in the robusta explants (Staritsky, 1970). Since then, many protocols using different kinds of explants were developed for different coffee species, including *C. Arabica* (Loyola-vargas et al., 2016). In coffee, the SE direct way is often described as a low frequency method and the indirect way as high frequency method (Etienne, 2005). In the low frequency process, only one medium is used and the embryos are obtained faster (approximately within 70 days) but in a smaller number, maximum 10 per explant. In the high frequency process, multiple media are used: (i) the callus induction medium, to provide the stimulus necessary for the cells to start dividing, (ii) the embryogenic callus production medium, were the callus will grow exponentially and (iii) the callus maintenance medium to further multiply the cells in exponential phase, select and keep the callus in the undifferentiated state and the embryo maturation and regeneration medium (van Boxtel and Berthouly, 1996) (**Table 1** and **Figure 2**). The firsts media to induce and produce embryogenic callus normally use a higher concentration of auxin, followed by a decrease or even complete removal of auxins, for embryo development (van Boxtel and Berthouly, 1996; Padua et al., 2014). In this methodology, the embryo production takes more time to develop (9–10 months), but hundreds of them can be obtained per gram of callus. It is possible to use a liquid medium for multiplication as well as for maturation. This is the preferred method for mass propagation since bioreactors can be used improving the embryo formation rates (Etienne, 2005). The use of semi-solid medium is less efficient, probably due to lower and less homogeneous diffusion of growth regulators on this type of medium which can lead to a lower concentration of auxins with increasing distance to the culture medium (van Boxtel and Berthouly, 1996; Etienne, 2005; Simões-costa et al., 2009).

**Table 1:** Medium composition for somatic embryogenesis in coffee developed by Berthouly and Michaux-Ferriere (1996) and the adaptations from Teixeira et al. (2004) (in italic) in mg/L and μM (growth regulators).

	Callus	Embryogenic	Callus	Embryo	Regeneration
	induction	callus	maintenance	maturation	medium
	medium	production			
Basic Medium	MS/2	MS/2	MS/2	MS/2	MS
Casein	100	200	100	400	–
Malt	400	800	200	400	–
Thiamine	10	20	5	10	10
Nicotinic acid (mg/L)	1	–	0.5	1	–
Pyridoxine (mg/L)	1	–	0.5	1	–
Myoinositol (mg/L)	100	200	50	200	100
Glycine (mg/L)	1	20	–	2	–
Cysteine (mg/L)	–	40	10	10	–
Adenine sulfate (mg/L)	–	60	–	40	–
2,4-D (μM)	2.26/*20*	4.52/*10*	4.52/*5*	–	–
IBA (μM)	4.92	-/*4.92*	-/*4.92*	-	-
2iP (μM)	9.84	-/*9.84*	-/*9.84*	-	-
BAP (μM)	-	17.76/-	-	17.76/*8.88*	1.33
NAA (μM)	-	-	-	*1,34*	-
KIN (μM)	-	-	4.65/-	-	-
Sucrose (mM)	88	88	44	88	117
pH	5.6	5.6	5.6	5.6	5.6
Phytagel (g/L)	2.5	2.5	-	-	-

*When there is only one information, it means they are the same in both protocols*.

### Factors Influencing SE in Coffee

#### Type of Explant

Somatic embryogenesis in coffee can be induced using different kinds of explants, but generally young leaves, which are available throughout the year, are the most commonly used. To obtain somatic embryos, many parameters need to be taken into account such as the physiological state of the mother plants, leaf age (young, mature, completely expanded), environmental growth conditions of the explant donor and even the month of collection, since it can affect the physiology of the mother plant (Molina et al., 2002; Santana et al., 2004). When the mother plants are kept in green houses, the influence of external conditions are less important since the environmental conditions are more controlled. However, the most determining factor for efficient SE in coffee remains the genotype (Molina et al., 2002; Etienne, 2005; Rezende et al., 2011). Molina et al. (2002) and Rezende et al. (2011) studied SE in different *C. arabica* genotypes showed that some genotypes are completely recalcitrant while in others 73% of formed callus was able to generate embryos.

#### Nutrients

The nutrient medium for SE may have different compositions, but the most commonly used is based on MS (Murashige and Skoog, 1962) and used in half-strength (Etienne, 2005; Gatica-Arias et al., 2008; Simões-costa et al., 2009). After the production of embryogenic callus, you can keep the cells in their undifferentiated state as callus, or the differentiation phase can be started in order to obtain the embryos (Etienne, 2005; Papanastasiou et al., 2008; Simões-costa et al., 2009). To keep the cells in an undifferentiated callus state, cell suspension cultures are commonly used. Stable embryogenic cell suspensions are obtained generally 2–3 months after their initiation in liquid medium. These cell suspensions are subcultured biweekly by renewing the culture media completely until the transfer to maturation medium and further plantlet regeneration. The cell suspension method has been applied with success for multiplication and short term maintenance of undifferentiated embryogenic calli for many species, including coffee (Etienne, 2005; Bobadilla Landey et al., 2015). The risk of somaclonal variation and the options to keep a good cell suspension are discussed further below.

#### Plant Growth Regulators

Like for all plant species, plant growth regulators (PGRs) play an essential role in coffee SE. The right balance between the kind of growth regulator and concentration is quite specific for each cultivar which makes the SE in coffee an empiric process (Santana-Buzzy et al., 2007). A big improvement on coffee SE was made with the implementation of 2 media, one for conditioning the explants and another for callus development (Sondahl and Sharp, 1977; Loyola-vargas et al., 2016). Nowadays, most of the protocols are based on the indirect protocol developed by Berthouly and Michaux-Ferriere (1996; van Boxtel and Berthouly, 1996; Etienne and Bertrand, 2003; Etienne, 2005; Gatica-Arias et al., 2008; Menéndez-Yuffá et al., 2010; Bobadilla Landey et al., 2015). During the years adaptations have been suggested by Teixeira et al. (2004) to improve the yield of embryos even further and have been applied (Padua et al., 2014; Silva et al., 2015; Campos et al., 2016). **Table 1** shows the original media described by Berthouly and Michaux-Ferriere (1996) and the adaptations made by Teixeira et al. (2004). Beyond the traditionally used PGRs (auxins and cytokines) other groups of growth regulators have been tested in established protocols. Ethylene proved to be successful to accelerate the embryogenic process and improve direct embryogenesis (Silva et al., 2011). Papanastasiou et al. (2008) induced SE and developed new plants from these embryos using only BA (6-benzyladenine) during the complete protocol. A new generation of growth regulators such as oligosaccharides, jasmonates, polyamines, and brassinosteroids has recently been studied to clarify their roles in SE, not only in coffee, but in other species (Jiménez, 2005; Ruduś et al., 2006; Yang and Zhang, 2010; Mira et al., 2016).

#### Growing Environment

Another very important factor that influences SE is the culture environment, for example, the gaseous concentration in the culture flasks. The concentration of dissolved CO_2_ or oxygen influences the development of somatic embryos. de Feria et al. (2003) tested two different concentrations of dissolved oxygen (50 and 80%) in the production of embryos from cell suspensions of *C. arabica* cv. Catimor. The total number of embryos and stage of maturation was significantly different in both concentrations. In the lower concentration, the number of embryos was lower but they were in a maturation more advanced stage. The opposite was observed in a concentration of 80% dissolved oxygen. This shows that the content of dissolved oxygen should be taken into account when developing a multiplication and regeneration protocol using bioreactors, since in this method you can provide additional ventilation. The effect of CO_2_ was also investigated by Barbón et al. (2008) in cell suspensions of coffee cv. Caturra rojo. A higher number of embryos was observed using low CO_2_ concentrations, suggesting that the CO_2_ concentration exerts a positive effect on the embryo growth when present in low concentrations in the medium, being toxic and blocking embryo development at higher concentrations (Barbón et al., 2008). A theory about how CO_2_ influences somatic embryo formation is through the pH alteration of the medium. In general the pH used to start a cell suspension is 5,7 ± 0,1. In the first days of culture there is a continuous decrease, reaching to pH 4, followed by an increase until almost the initial level. The moment when the pH is increasing again as said to coincide with the first embryo formation (de Feria et al., 2003; Barbón et al., 2008). Changes in pH seems to be an important factor in the regulation of SE and can be controlled naturally by the growth and normal substances release from the cells (Chung et al., 2006, 2016). The cell density also influences SE. A high density of cells leads to multiplication of embryogenic cells instead of development of embryos. Embryo maturation thus benefits from a low density (Santana et al., 2004; Etienne, 2005). This fact is not only observed in coffee and happens probably due to a production of some conditioning substances such as 4-hydroxybenzyl alcohol that inhibits the embryo formation (Kobayashi et al., 2000; Santana et al., 2004).

Although these studies show the individual importance of certain physical conditions in the production of SE, we should not forget that during *in vitro* cultivation all these parameters act together, and influence each other, like the pH and the amount of CO_2_ in the medium. The way how they interact can help to improve or predict the development of SE.

#### Somaclonal Variation

Plants regenerate from tissue culture are expected to have identical genetic material to the mother plants and thus, keep their intrinsic characteristics. However, differences in the phenotype and/or genotype of plants from tissue culture are often observed and called somaclonal variation (SV). SV can be caused by point mutations, transposon activity, chromosomal rearrangements, or ploidy level changes. It happens during the extensive cell division probably due to stress conditions such as wounds, exposure to hormones and/or specific compounds in the growth media (Azma et al., 2013; Bobadilla Landey et al., 2015). In many tissue culture procedures that involve dividing cells, the risk of somaclonal variation and contamination due to the frequent manipulations is still high. To reduce this risk it is preferred not to maintain suspensions or calli for more than 6 months (Etienne, 2005; Bobadilla Landey et al., 2015). Somaclonal variation among coffee plants regenerated through somatic embryogenesis ranges from 0 to 93% and depends on the genotype, explant source, culture age, type and concentration of plant growth regulators in the medium (Etienne and Bertrand, 2001; Etienne et al., 2002; Gatica-Arias et al., 2008; Bobadilla Landey et al., 2013, 2015). *In vitro* culture studies showed that coffee plants directly regenerated from up to 4-month-old embryogenic calli or cell suspensions present low (1,3%) or even 0 somaclonal variation rates while a gradual increase of SV was observed with increasing age (6, 10, and 25% in plants produced from cell suspensions aged 6, 9, and 12 months, respectively) (Etienne and Bertrand, 2003; Bobadilla Landey et al., 2013, 2015). This rate can reach 93% after 27 months (Bobadilla Landey et al., 2015). According to Bobadilla Landey et al. (2015), genetic polymorphisms and epigenetic changes are particularly limited during cell culture aging, while aneuploidy plays a major role in SV, indicating that mitotic aberrations play a major role in somaclonal variation *C. arabica.* SV can be detected by phenotyping in combination with the use of molecular markers (see below). Cryopreservation techniques has been applied not only in coffee, but for many species in order to store cell lines of interesting genotypes, species that not tolerate the conventional way of storage or threatened species. In the specific case of SE, it can be applied to keep good quality embryogenic cell suspensions without the risk of losing them because of somaclonal variation, loss of regeneration capacity or contamination (Dussert et al., 1997). Cryopreservation provides a safe way to store genetic material for undetermined time.

## The Potential of Omics Technology and Molecular Markers to Improve SE

### Genes Involved in SE

In the past, studies about SE were mainly empirical and focused on establishing and optimizing somatic embryo production in different species, much more than to understand the mechanisms behind this event. Even though different studies investigated the cellular and molecular changes during SE in many different plant species, the molecular basis of the factors involved in initiation of SE process are not completely understood (Yang and Zhang, 2010; Mukul-lópez et al., 2012; Guzmán-García et al., 2013). The Brazilian Coffee Genome Project sequenced 214.964 ESTs (Expressed Sequence Tags) belonging to 33.000 different genes from 37 cDNA libraries of leaves, roots, flowers, fruits, embryogenic calli and zygotic embryos of *C. arabica, C. canephora*, and *C. racemosa*. Those tissues were collected under different physiological states as different stages of fruit development and flowering, young and mature leaves and under influence of biotic and abiotic stress (Esteves Vieira et al., 2006). The large number of data generated by this consortium is extremely valuable to help to clarify molecular events like SE (Silva et al., 2013). Recently the genome of *C. canephora* was published, opening new possibilities to explore and understand the coffee diversity (Denoeud et al., 2014). We also published the proteome profile of embryogenic calli, a first step toward insight into totipotency (Campos et al., 2016). The main challenge now is to integrate all the generated knowledge about coffee physiology to elucidate processes such as somatic embryogenesis. It is already known that the expression of some genes are linked to different stages of SE: BABY BOOM1 (BBM), LEAFY COTYLEDON 1 and 2 (LEC1/LEC2), WUSCHEL-RELATED HOMEOBOX (WUS) and SOMATIC EMBRYOGENESIS RECEPTOR KINASE (SERK) (Hecht et al., 2001; Arroyo-Herrera et al., 2008; Yang and Zhang, 2010; Yang and Karlson, 2011; Ma et al., 2012; Nic-Can et al., 2013). Since these four genes are relatively well known in other species, especially in model organisms, their role and specifically presence or similarity in coffee are further discussed. Silva et al. (2013) identified 15 EST-contigs related to the embryogenesis process in coffee. Among those 15 ESTs, 1 sequence was annotated to abiotic stress. 9 EST-contigs could not be identified in the EST database generated by the Brazilian Coffee Genome Project, but were detected only in their own embryogenic material. The other 5 ESTs could be readily associated with coffee embryogenesis showing similarities with proteins as polygalacturonase, cysteine-proteinase, expansine, allergenine, and WUS.

#### Baby Boom (BBM)

Baby boom is known to be expressed in developing embryos and is correlated to cell proliferation and morphogenesis (Boutilier et al., 2002; Nic-Can et al., 2013; Silva et al., 2015). Boutilier et al. (2002) studying *Brassica napus*, published one of the first reports linking BBM1 to the induction of embryogenesis. They showed that the overexpression of BBM has a role in promoting cell proliferation and morphogenesis during embryogenesis. The BBM products are similar to the AP2/ERF family of proteins, that are specific plant proteins playing a role in many biological processes such as determination of cell identity in leafs and floral organs, response to biotic and abiotic stress, embryogenic processes and cellular proliferation in meristematic regions (Riechmann and Meyerowitz, 1996; Boutilier et al., 2002). Specifically in coffee, Silva et al. (2015), showed higher expression levels of two BBM homologous (similarity with *e*-value > 10^-4^) sequences in embryogenic calli and cell suspensions when compared to non-embryogenic calli. These authors proposed that BBM homologs found in *C. arabica*, termed as CaBBM, could be used as a molecular markers to assist in the optimization of the regeneration process more specifically to determine the optimal time to start cell suspensions lines from embryogenic calli (CaBBM isoforms are highly expressed). With the help of additional markers it would thus become possible to detect and select in advance, cell suspensions with a higher embryogenic capacity.

#### Leafy Cotyledon (LEC)

The gene Leafy Cotyledon was first identified and characterized in Arabidopsis (Lotan et al., 1998). Its expression is crucial for the cotyledon identity formation and the completion of embryo maturation (Lotan et al., 1998). Ectopic expression of LEC in vegetative tissues suggests its importance for cellular differentiation and embryo morphogenesis through the induction of somatic embryo formation. LEC1 is expressed more profusely during the seed maturation and integrates different activities in both, ZE (zygotic embryo) and SE to induce the embryogenic program and maturation (Lotan et al., 1998; Boutilier et al., 2002; Braybrook and Harada, 2008; Nic-Can et al., 2013). Nic-Can et al. (2013) studied the expression of this gene in coffee. The authors suggested that LEC expression is essential for embryo maturation since its expression was observed only after SE induction. Regulation of this genes was proven to be influenced by epigenetics (see below).

#### Somatic Embryogenesis Receptor Kinase (SERK)

The gene SERK was first described in carrot (dcSERK) cell suspensions (Schmidt et al., 1997). Its expression is associated with the early stages of embryo development. Based on its expression pattern, Hecht et al. (2001) suggested to use SERK as a marker for embryogenic competence. The same expression pattern was found during early zygotic embryogenesis, proving the similarity of both types of embryogenic development (Schmidt et al., 1997). Homologs of SERK were identified in different species as maize, cacao, rice, sunflower, and citrus (Baudino et al., 2001; Thomas et al., 2004; de Oliveira Santos et al., 2005; Shimada et al., 2005; Ito et al., 2005) and showed a role in the SE of these species. However, in rice, it showed also constitutive expression (Ito et al., 2005). The dcSERK gene encodes a Leu-rich repeat (LRR) transmembrane receptor-like kinase (RLK). This transmembrane receptor plays a role in many biological processes as receptor of growth regulators, maintenance of balance between undifferentiated cells and cells designated to form new organs in meristematic regions (Schmidt et al., 1997; Hecht et al., 2001). This gene was overexpressed in Arabidopsis and its importance to acquire embryogenic competence has been studied (Hecht et al., 2001; Yang and Karlson, 2011; Ma et al., 2012). Although SERK homologs can show constitutive expression in other crops like rice, maize and *Vitis vinifera* (Baudino et al., 2001; Ito et al., 2005; Schellenbaum et al., 2008), in species like Arabidopsis, pineapple and carrot, a higher expression in embryogenic material was observed compared to non-embryogenic (Schmidt et al., 1997; Hecht et al., 2001; Ma et al., 2012). The presence of a SERK ortholog (CaSERK) in embryogenic cell suspensions of coffee was confirmed by Silva et al. (2014). The authors identified *in silico*, 18 EST-contigs similar to the SERK family from the database generated by the Brazilian Coffee Genome Project. Among these 18, one paralog showed high similarity with SERK domains and showed *in silico* expression only in embryogenic material. The exclusive expression of CaSERK in embryogenic material of coffee suggests a role in the embryogenic capacity.

#### Wuschel-Related Homeobox (WUS)

Wuschel-related homeobox is a transcription factor of the family Wuschel-related homeobox that is expressed during zygotic embryogenesis in Arabidopsis. WUS is also known to keep cells in an undifferentiated phase (Arroyo-Herrera et al., 2008), plays a specialized function in the embryo development by maintaining the vascular procambium and is highly expressed during germination of zygotic embryos. In *C. canephora*, a heterolog of WUS was capable to promote the induction of somatic cells (Arroyo-Herrera et al., 2008; Silva et al., 2013) and its overexpression increased 400% the production of somatic embryos (Arroyo-Herrera et al., 2008).

#### Epigenetic Regulation of Genes Plays a Role in SE

Nic-Can et al. (2013) reported a crosstalk between DNA methylation and histone modifications during the earliest embryogenic stages of SE using Chromatin Immunoprecipitation assays. They prove that the genes LEC1, BBM1 and WUS related gene WOX4 are under epigenetic control in relation to the embryogenic capacity in *C. canephora*. An increase in global DNA methylation was observed during the initial phase of induction of SE followed by a decrease during the course of maturation in embryos and ending in an increased DNA methylation when the first stage (globular) of embryo development appears at day 56. The comparison of the different stages of embryo development in SE and ZE, and *in vitro* plantlets indicated that the embryogenic differentiation is linked with DNA methylation. Histone methylation was also identified as a factor of influence (Bobadilla Landey et al., 2013; Nic-Can et al., 2013).

### Proteomics Linked to SE

Studies in plant biology trough proteomics have increased considerably in the recent years. The main cause of this growing interest is that proteomics provides an overview of the metabolism, complementary to the genomics results (Palma et al., 2011). It is known that the correlation between mRNA and protein at the same moment of extraction is often low (Jansen et al., 2002; Carpentier et al., 2008). High throughput proteomics for non-model plants has been used to solve this problem and generate more applicable results (Carpentier and America, 2014). Compared to transcriptomics, the proteomics approach has a great potential to study non-model plants since DNA sequences are less conserved as amino acid sequences being a promise alternative to understand plant metabolism (Zivy et al., 2015). This is specifically valid for *C. arabica* of which the genome is not sequenced yet but from which RNA-seq data are available. The sequence of *C. canephora* genome is also a really helpful tool that can be used to extrapolate for *C. arabica* and provide good results for plant understanding and coffee breeding. Proteomics studies on SE have already been executed in some species such as *V. vinifera* (Marsoni et al., 2008; Croce et al., 2009), cassava (Baba et al., 2008; Li et al., 2010), cacao (Noah et al., 2013), avocado (Guzmán-García et al., 2013), maize (Varhaníková et al., 2014), sugarcane (Heringer et al., 2015), and coffee (Tonietto et al., 2012; Campos et al., 2016). We identified 1052 non-redundant proteins, being 5 annotated to embryogenic capacity (Campos et al., 2016). Many proteins are without any annotation and so are still uncharacterized, showing the poor molecular knowledge about SE in coffee. Our publication of the proteome of somatic calli in coffee can be considered as the initial step to understand the embryogenic process in coffee and the annotation of proteins can guide further investigations in this topic. Most of these studies focus on differences between embryogenic and non-embryogenic calli. Some proteins specifically appear in embryogenic and not in non-embryogenic cell lines and proposed some molecular markers for different stages of SE as enolase and globulin S11 for torpedo stage of embryo development (Lippert et al., 2005; Tonietto et al., 2012). Tonietto et al. (2012) identified 14 proteins in coffee linked to the different phases of embryo development during SE. The most abundant were proteins related to energy production. The need for more energy is compatible with the major cell division that follows these stages of development (Baba et al., 2008). Correlation to specific proteins could be found for each embryo development stage (globular, torpedo, and cotyledonar). Proteins related to stress response were also identified, such as HEAT SHOCK PROTEIN (HSP 70) and cytoplasmic aldolase being more abundant in the cotyledonary stage. Stress is known to be an important factor to induce somatic embryogenesis and the presence of these proteins corroborate to this fact (Fehér et al., 2003; Tonietto et al., 2012; Fehér, 2015).

## Critical Reflections and Practical Applications of SE in Coffee

Obviously, many differences in the proteome and/or transcriptome/ metabolome between cell types coming from diverse differentiated tissues have been observed. Cells coming from various tissues of a differentiated embryo are physiologically very different from a callus. The key question is whether differences in protein abundance or its presence/absence can be linked to embryogenic capacity. The strength of molecular markers is the prediction of the embryogenic capacity of calli before they have differentiated into embryos and to use this information to steer current the protocols. For this, the right experimental setup is crucial where the comparison is performed before the cells differentiated into different tissues. A potential experimental setup might be to sample a part of the cloned calli where the cytological characteristics are identical or differences at least not observable. The other part is used to complete the differentiation procedure and evaluate the embryogenic potential of each sample batch. For the interesting batches, integrated omics analysis needs to be done and correlations can be sought between transcripts, proteins or metabolites and the observed embryogenic capacity. Although the expression of several genes is proven in coffee embryogenic cultures, the specific role and regulation is still not known for many of them.

One important application of SE is the possibility to accelerate breeding programs through genetic manipulation and rapid multiplication rate. This technology can also be applied for mass propagation on an industrial scale beside the maintenance of genetic resources (Simões-costa et al., 2009). Conventionally *C. arabica* is propagated trough seeds. Normally, after a breeding process (at least 20 years), the seed lines are considered pure and are sold like that. Coffee’s seeds are considered to be non-orthodox, which means they support partial dehydration but they can not be stored for long periods in conventional gene banks at -20°C (Simões-costa et al., 2009). Cryopreservation of seeds is a good alternative, showing lower maintenance costs compared to field or *in vitro* conservation (Dussert et al., 1997; Dulloo et al., 2009). Propagation using cuttings is available only for *C. canephora*. In Arabica coffee, the multiplication rates by cuttings are still unsatisfactory, being not commercially used. Somatic embryogenesis for *C. arabica* has been applied on a commercial scale in Central America. Up to date around 7 millions of plantlets derived from SE are grown in Central American Fields (Etienne et al., 2016). Since 2006 a production unit in Nicaragua (ECOM Trading) and another one in Xalapa (Mexico) has been set up. However, the cost-effectiveness of SE for commercial coffee propagation is still unsatisfactory, being around 2 dollars/plantlet while the conventional propagation cost is 0,35 dollars (Bertrand et al., 2012). It happens mainly due to two reasons, there is a lot of plant losses during acclimation phase and plants regenerate from SE are still not productive enough due to the some differences: plantlets from SE have a smaller hypocotyl, a reduced leaf area, atrophied cotyledons and a poor development of roots (Bertrand et al., 2012; Etienne et al., 2013). Recently, Georget et al. (2017) showed the possibility to multiply coffee SE plantlets via horticultural rooted mini-cutting (HRMC). With this technique the production drops considerable, facilitating the use of SE in an industrial scale.

## Conclusion and Future Perspectives

As mentioned before, one of the principal bottlenecks for the industrial use of SE in coffee is the conversion phase from embryo to plants, together with the low embryo formation rate in *C. arabica* cultivars. The use of temporary immersion bioreactors is normally used for the upscaling and optimization of the conversion phase (Etienne et al., 2006; Gatica-Arias et al., 2008). In general the proportion of torpedo embryos obtained in a conventional culture in Erlenmeyer flasks turns around 20–30% and in the immersion bioreactors the proportion was up to 90% (Etienne-Barry et al., 1999; Etienne et al., 2006). Embryo formation is also time consuming, and bioreactors also help to shorten the period. While in an Erlenmeyer flasks it takes normally 6 months, with a bioreactor this time can drop to 6 weeks (Gatica-Arias et al., 2008). Georget et al. (2017) found a new and more efficient way to propagate SE plantlets. The horticultural rooted mini-cuttings, although efficient, can be improved via a more uniform embryo formation. The age of SE plantlets harvested from the nursey is an important factor for the successful rooting and growing. Molecular studies are important for this point. The knowledge of molecular changes during this process and the possibility to manipulate these factors can improve the embryo formation and development at the same time. Culture conditions like *in vitro* multiplication and the early growing conditions of the SE plantlets can also be optimized for this purpose.

Another challenge in coffee SE is the early identification of cells with good embryogenic capacity. The first classification of the embryogenic quality is made based on morphological characteristics, this parameter can lead to false positives since some cells can appear to have a good embryogenic capacity but after a while, they don’t regenerate any embryos or a really small number. Early identification would avoid the maintenance of these cells, saving money, time and work. Again, the molecular knowledge is indispensable for this. Gene expression related to SE induction or embryo maturation can be used as markers for this early identification. Universal markers are really difficult to detect. A solution for this would be the integration from genomics, transcriptomics, proteomics, metabolomics and morphology for the identification of good cells or suspensions.

The large amount of protocols available in the literature proves that coffee is not a recalcitrant species for SE. However, a big range of variability to successful regeneration has been reported. Too many different protocols exist and too many cultivars show a differential outcome to the same protocol. A better understanding of the processes as a whole will clarify what are the current specific bottlenecks and what are the solutions. Molecular markers are key to improve the whole process of SE and the step toward full commercialization. Moreover it will generate a better knowledge about embryogenesis and totipotency in higher plants, an important biological phenomenon still not well understood.

The early selection of good quality cell suspensions through molecular markers could avoid losing time in maintaining and regenerating material that has none or has low embryogenic potential. As we showed is difficult to have universal individual markers. Only the combination of several markers can be conclusive. This fact also shows the complexity of totipotency and the embryogenic process. Although the basis can be similar, each species presents its own specificity and requirements for embryo development. For increasing the practical applications of SE in coffee, more research, but specially the integration of existing studies and results is necessary.

The most accepted theory about the origin of somatic embryogenic cells is that somatic cells can regain embryogenic capacity (when the right stimulus is applied) by passing through a process of dedifferentiation. We presented an alternative theory; i.e., plants maintain some cells in the totipotent state (embryogenic) in their meristematic tissues; these cells could be considered as plant stem cells. Most cells do not respond to the stimulus because they do not have the capacity to do so. Only a few cells keep this capacity. The stimulus just “wake up” this cells. We would like to open this discussion to the plant scientific community.

## Author Contributions

The authors jointly wrote the paper. NC is an expert in coffee embryogenesis. BP is the main mind behind the totipotency theory. SC behind the integration of omics technologies.

## Conflict of Interest Statement

The authors declare that the research was conducted in the absence of any commercial or financial relationships that could be construed as a potential conflict of interest.
